# Characterization of indeterminate renal masses with molecular imaging: how do we turn potential into reality?

**DOI:** 10.1186/s13550-017-0277-0

**Published:** 2017-04-12

**Authors:** Steven P. Rowe, Mehrbod S. Javadi, Mohamad E. Allaf, Michael A. Gorin

**Affiliations:** 1 Division of Nuclear Medicine and Molecular Imaging, The Russell H. Morgan Department of Radiology and Radiological Science, Johns Hopkins University School of Medicine, 600 N. Wolfe St., Baltimore, MD 21287 USA; 2 The James Buchanan Brady Urological Institute and Department of Urology, Johns Hopkins University School of Medicine, Baltimore, MD USA

**Keywords:** Small renal mass, Renal cell carcinoma, Oncocytoma, SPECT/CT, ^99m^Tc-sestamibi

## Background

The majority of enhancing renal masses are unable to be effectively characterized as benign or malignant using standard cross-sectional imaging modalities including multi-phase computed tomography (CT) [1] and magnetic resonance imaging (MRI) [2]. This is particularly unfortunate in light of the steady increase in the incidence of these lesions, many of which are discovered incidentally on imaging studies performed for non-urologic indications [3, 4]. Moreover, it has been estimated that upwards of 5600 unnecessary partial and radical nephrectomies are performed each year in the USA for the false presumption of cancer [5]. In light of these data, recently, there has been a growing interest in the use of molecular imaging to characterize the aggressiveness of renal masses [6, 7].

To date, several reports have been published on the ability of ^99m^Tc-sestamibi planar and single photon emission computed tomography (SPECT)/CT imaging to differentiate mitochondrial-rich benign and indolent renal masses such as oncocytomas and hybrid oncocytic/chromophobe tumors (HOCTs) from more aggressive renal tumor histologies including the clear cell subtype of renal cell carcinoma (RCC) [8–10]. In this issue of *European Journal of Nuclear Medicine and Molecular Imaging Research*, Tzortzakakis et al. [11] add to this body of literature with a prospective study evaluating ^99m^Tc-sestamibi SPECT/CT in 24 patients with 31 clinically localized T1 renal masses planned for biopsy or surgery. The authors reported that 11 of 12 (92%) oncocytomas and 3 of 3 (100%) HOCTs demonstrated ^99m^Tc-sestamibi uptake, while all other lesions were negative for uptake with the exception of mild uptake in a single papillary RCC. These findings are concordant with earlier studies [9, 10] and dramatically increase the number of ^99m^Tc-sestamibi positive oncocytomas and HOCTs reported in the literature.

As a brief aside, it should be mentioned that there is also a growing interest in the use of renal mass biopsy for determining the histology of renal tumors, particularly as data from two recent systematic reviews have suggested that the rates of complications and non-diagnostic biopsies are tolerable [12, 13]. However, limitations of renal mass biopsy remain, including the intrinsically invasive nature of this procedure, the inaccessibility of some tumors to safe approaches for biopsy, and the heterogeneous population of renal masses that can present as oncocytic neoplasms on biopsy [14, 15]. As such, the non-invasive characterization of renal masses with molecular imaging techniques continues to be of interest and may serve as an adjunct to, or even a replacement of, biopsy in selected cases.

## Additional prospective studies

The MIDOR (Molecular Imaging for Differential Diagnosis of Oncocytoma from Renal Cell Carcinoma) Trial, from which the manuscript by Tzortzakakis et al. [11] reports initial results, is the first report in the literature on the implementation of ^99m^Tc-sestamibi SPECT/CT outside of Johns Hopkins Hospital in Baltimore, MD, USA. Additional prospectively collected data are critical for validating the robustness of this imaging test across centers, scanners, and interpreting imaging experts. Ultimately, we can expect that the pearls and pitfalls of using ^99m^Tc-sestambib SPECT/CT for renal mass characterization will become most apparent with increased patient numbers. Additionally, larger datasets are required to ensure that all renal tumor histologies (some of which are quite rare) are studied in sufficient numbers to establish the various patterns of ^99m^Tc-sestamibi uptake.

## Development of improved quantitative methods

SPECT imaging has long been considered a qualitative modality; however, new methods for attenuation correction, scatter correction, compensation for distance-dependent blurring/collimator-detector response, and partial volume correction have allowed for true quantitative SPECT to be performed [16]. While both Rowe et al. [9] and Gorin et al. [10] utilized a semi-quantitative method to describe the uptake of ^99m^Tc-sestamibi by renal masses, the method used in these reports (ratio of the highest count voxel in the mass to the highest count voxel in the ipsilateral renal parenchyma) is subject to potential error. Moreover, the images that were used for this analysis were from standard clinical non-quantitative acquisitions.

As Tzortzakakis et al. [11] astutely point out in the “Discussion” section of their manuscript that quantitative methods may be of importance for optimal characterization of renal masses with ^99m^Tc-sestamibi SPECT/CT. Although a visual analysis of renal masses imaged with ^99m^Tc-sestamibi SPECT/CT will always be an important part of interpreting these studies, a numerical threshold (as has been proposed by Gorin et al. [10]) may be a useful interpretational adjunct. However, determination of true SPECT standardized uptake values is likely to be a more reliable means of characterizing these tumors. Thus, further investigation of quantitative SPECT methodologies is needed.

## Pipeline of new radiotracers

The promise of ^99m^Tc-sestamibi SPECT/CT must be tempered by the fact that an imaging test that uses a single radiotracer can provide only limited information for characterizing indeterminate renal masses. Indeed, while ^99m^Tc-sestamibi uptake appears to allow for the reliable identification of benign/indolent oncocytomas and HOCTs [9–11], not all masses that fail to accumulate ^99m^Tc-sestamibi will behave in an aggressive manner (e.g., chromophobe RCC and low-grade papillary RCCs). As such, the use of other radiotracers in addition to ^99m^Tc-sestamibi may allow for more complete risk stratification. Already, the radiolabeled monoclonal antibody ^124^I-girentuximab has been used in a phase III clinical trial to identify clear cell RCCs using positron emission tomography (PET) imaging through binding to carbonic anhydrase IX (CAIX), a cell surface enzyme that is not expressed in normal renal tissue or by other renal tumor histologies [17]. New small molecule SPECT and PET radiotracers that target carbonic anhydrase IX are in preclinical development (for example [18] and [19]). The SPECT agent described in Yang et al. [18] is of particular interest given its labeling with ^111^In which would allow for dual radiotracer SPECT in combination with ^99m^Tc-sestamibi.

Although lacking dual radiotracer capability, PET does have advantages over SPECT including established methods of quantitation and superior spatial resolution. As such, the development of PET radiotracers for renal mass characterization should be of significant interest to the field. As noted above, a CAIX PET radiotracer (^124^I-girentuximab) has already been extensively studied and may prove clinically useful in the future [17]. Furthermore, mitochondrial PET imaging agents have been described for cardiac and non-cardiac applications [20, 21] and deserve to be explored for their potential utility in identifying mitochondrial-rich renal masses such as oncocytomas and HOCTs.

## Conclusions

The work of Tzortzakakis and coworkers [11] is a critical step in laying the foundation for the widespread use of molecular imaging for renal mass characterization, although further efforts would be of value for advancing the field to the point of routine clinical application. Additional prospectively collected data as well as research in quantitative imaging methods and the development novel radiotracers could usher in an era of non-invasive renal mass characterization. The refinement of molecular imaging techniques for renal mass characterization should be a priority for nuclear medicine, as this has the potential to greatly benefit patients by sparing many of the morbidity of invasive procedures in addition to unnecessary renal surgery.

## Abbreviations

CAIX: Carbonic anhydrase IX
CT: Computed tomography
HOCT: Hybrid oncocytic/chromophobe tumor
MIDOR: Molecular Imaging for Differential Diagnosis of Oncocytoma from Renal Cell Carcinoma
MRI: Magnetic resonance imaging
PET: Positron emission tomography
RCC: Renal cell carcinoma
SPECT: Single photon emission computed tomography
